# VTE prophylaxis admission assessment full cycle audit and QI project

**DOI:** 10.1192/bjo.2021.244

**Published:** 2021-06-18

**Authors:** Krystyna Drewniak, Mark Fielding

**Affiliations:** Dane Garth, Dova Unit

## Abstract

**Aims:**

The aim of the project was to assess completion rates for the VTE prophylaxis assessment for patients admitted to Dova Unit, Dane Garth. Another aim of the project was to identify areas for improvement and changes which could increase compliance rates.

**Method:**

In the first cycle of the audit 20 randomly selected patients admitted to Dova Unit, Dane Garth between June and December 2020 were identified and included in the project. Data were then collected from the online patient record system Rio and analysed using an excel spreadsheet.

In the second cycle of the audit 10 randomly selected patients admitted to Dova Unit, Dane Garth between January and February 2021 were identified and included in the project. Data were then collected from the online patient record system Rio, analysed using an excel spreadsheet and compared with the results obtained in the first cycle of the project.

**Result:**

In the first cycle of the audit the overall compliance was found to be 35%. VTE Risk assessment was completed for 50% of patients included in the study. ‘Active VTE on admission’ section of the VTE prophylaxis admission assessment was completed for 30% of patients included in the study. ‘Active VTE at 72 hours’ section was completed for 20% of the patients in the study and the ‘risk assessment for VTE' form was completed for 40% of patients included in the study.

In the second cycle of the audit the overall compliance was found to be 50%. VTE Risk assessment was completed for 60% of patients included in the study. ‘Active VTE on admission’ section of the VTE prophylaxis admission assessment was completed for 40% of patients included in the study. ‘Active VTE at 72 hours’ was completed for 40% of the patients included in the study and ‘risk assessment for VTE' form was completed for 60% of patients included in the study.

**Conclusion:**

There was an overall improvement in the completion rates for the VTE prophylaxis admission assessment as a result of conducting the project. Working with the junior doctors and other healthcare professionals responsible for completing the VTE prophylaxis admission assessment, we aim to improve our completion rates of vital information even further.

